# Sampling forests with terrestrial laser scanning

**DOI:** 10.1093/aob/mcab073

**Published:** 2021-06-10

**Authors:** Peter B Boucher, Ian Paynter, David A Orwig, Ilan Valencius, Crystal Schaaf

**Affiliations:** 1 School for the Environment, University of Massachusetts, Boston, MA, USA; 2 Department of Organismic and Evolutionary Biology (OEB), Harvard University, Cambridge, MA, USA; 3 Universities Space Research Association (USRA), GESTAR, NASA Earth Sciences, GSFC, Greenbelt, MD, USA; 4 Harvard Forest, Harvard University, Petersham, MA, USA

**Keywords:** Terrestrial LiDAR scanning, TLS, forest inventory, forest survey, timber cruise, forestry, sampling

## Abstract

**Background and Aims:**

Terrestrial laser scanners (TLSs) have successfully captured various properties of individual trees and have potential to further increase the quality and efficiency of forest surveys. However, TLSs are limited to line of sight observations, and forests are complex structural environments that can occlude TLS beams and thereby cause incomplete TLS samples. We evaluate the prevalence and sources of occlusion that limit line of sight to forest stems for TLS scans, assess the impacts of TLS sample incompleteness, and evaluate sampling strategies and data analysis techniques aimed at improving sample quality and representativeness.

**Methods:**

We use a large number of TLS scans (761), taken across a 255 650-m^2^ area of forest with detailed field survey data: the Harvard Forest Global Earth Observatory (ForestGEO) (MA, USA). Sets of TLS returns are matched to stem positions in the field surveys to derive TLS-observed stem sets, which are compared with two additional stem sets derived solely from the field survey data: a set of stems within a fixed range from the TLS and a set of stems based on 2-D modelling of line of sight. Stem counts and densities are compared between the stem sets, and four alternative derivations of area to correct stem densities for the effects of occlusion are evaluated. Representation of diameter at breast height and species, drawn from the field survey data, are also compared between the stem sets.

**Key Results:**

Occlusion from non-stem sources was the major influence on TLS line of sight. Transect and point TLS samples demonstrated better representativeness of some stem properties than did plots. Deriving sampled area from TLS scans improved estimates of stem density.

**Conclusions:**

TLS sampling efforts should consider alternative sampling strategies and move towards in-progress assessment of sample quality and dynamic adaptation of sampling.

## INTRODUCTION

Forest surveys seek to estimate, for large spatial areas of forest, attributes that are of commercial or ecological interest, or of relevance to ecosystem services, such as stem count, species composition, tree height or timber volume (Wenger, 1984). Typically, forest surveys achieve a wide spatial extent for their estimates of forest attributes by collecting measurements from individual trees in selected sample areas, treating these observations as representative of a larger area of interest. Therefore, there are two major contributing factors to the quality of forest surveys: the accuracy of measurements of forest attributes and the representativeness of sampled areas to the wider area of interest. This study provides quantitative assessments of the challenges faced in improving each of these factors for forest surveys conducted with terrestrial laser scanners (TLSs) and evaluates potential improvements in sampling strategies and data processing techniques to meet these challenges.

Terrestrial laser scanners utilize light detection and ranging (LiDAR) technology to capture the 3-D structure of their surroundings as a collection of returns resulting from emitted pulses of energy reflecting from objects. Many studies have sought to evaluate and improve the accuracy of TLS estimates of specific forest attributes that have historically been retrieved by other methods (Strahler *et al.*, 2008; Yao *et al.*, 2011; Newnham *et al.*, 2015; Disney *et al.*, 2018; Calders *et al.*, 2020). For example, the ability of TLSs to retrieve diameter at breast height (DBH) has been compared with observations made using relascopes, dendrobands and diameter tapes (Strahler *et al.*, 2008; Yao *et al.*, 2011). The accuracy of TLS estimates of forest attributes has compared favourably to established field methods in some studies (Lovell *et al.*, 2011; Yao *et al.*, 2011; Calders *et al.*, 2015), and TLSs are anticipated to have an ever-increasing role in forest surveys (Danson *et al.*, 2018; Calders *et al.*, 2020).

As the resolution, accuracy and information content of TLS data are continuing to improve, TLSs can expand sampling to improve representativeness, and capture novel and useful attributes of forests. However, a deeper understanding of the uncertainties arising specifically from the application of TLS technology to forest surveys is still required (Strahler *et al.*, 2008; Lovell *et al.*, 2011; Calders *et al.*, 2014; Newnham *et al.*, 2015; Morsdorf *et al.*, 2018; Disney *et al.*, 2018), and further refinement of the design and implementation of sampling strategies for TLS surveys of forests is also needed (Calders *et al.*, 2014; Abegg *et al.*, 2017; Wilkes *et al.*, 2017).

The most obvious challenge to TLS forest surveys is that TLS observations are inherently limited to line of sight. Therefore, a targeted tree (or a targeted component of a tree such as the stem) might not be observed because of occlusion by other trees, other vegetation, other objects or topography (Strahler *et al.*, 2008; Lovell *et al.*, 2011; Morsdorf *et al.*, 2018; Schneider *et al.*, 2019). This tendency becomes a problem when there are predefined bounds for an intended sample, for example a square plot, and therefore the assumption is that all of the components of interest inside the sample will be observed. For example, the area used to calculate an estimate of stem density would be the extent of the predefined sample, and if not all of the stems are captured in the TLS observation, then the resulting stem density would be an underestimate (less stems, divided over the same area). Similarly, if a sample is an individual TLS scan, the default assumption would be that its coverage is a circle with a radius equal to the maximum range (or maximum effective range) of the TLS (Paynter *et al.*, 2018b), but only components within the range and the line of sight of the TLS will be observed.

Standard protocols for manual timber inventory operate similarly to a single TLS scan, documenting all trees within a range from a fixed position, but these protocols instruct surveyors to move from their fixed sampling position to assess trees that were previously obscured in the surveyor’s line of sight (Wenger, 1984). We can understand this movement to be an effort to complete the sample, and we can understand how an incomplete sample would be a source of uncertainty. In many TLS forest surveys, considerable effort is put into attempting to complete samples by scanning multiple times within the predefined sample area (Wilkes *et al.*, 2017). For example, in the commonly used square plots, TLS scans are typically conducted in a grid pattern, and this approach usually has a hypothetical coverage that is highly redundant, given the TLS instrument’s range and angular resolution, and the distance between the scans. However, even with the investment of a high level of sampling effort, it has been suggested that extreme patterns of occlusion can still persist and result in incomplete samples (Morsdorf *et al.*, 2018; Paynter *et al*., 2018*a*, *b*).

In this study, we quantitatively explore expectations for TLS sample completeness and the uncertainties caused by incomplete samples in a large number of TLS scans (761), conducted with plot, transect and point-based sampling strategies in the Harvard Forest ForestGEO site. We look at whether the use of particular sampling strategies (Abegg *et al.*, 2017) or *a posteriori* assessment of TLS sample coverage (deriving the observed area from the TLS scan data) might mitigate these challenges. We also evaluate whether different sampling strategies offer improvements in quality and efficiency for retrieving representative samples of forest properties for the wider area of interest.

It should be noted that the accuracy of retrieving any particular forest property with TLS is heavily dependent on the specifications of the instrument and the retrieval method for the property (Wilkes *et al.*, 2017; Danson *et al.*, 2018; Disney *et al.*, 2018). However, this study focuses solely on sample completeness as an influence on property retrieval accuracy. We avoid the potential influences of TLS specifications and property retrieval methods by utilizing the detailed ForestGEO field survey data, which include every stem with a DBH >1 cm, for the locations where the TLS scans were conducted. Stem presence, position, DBH and species are taken directly from the ForestGEO field survey data, and the TLS scans are assessed only for whether they had line of sight to the stems. Therefore, this study is not an evaluation of the operational capabilities of a particular TLS instrument, or a particular forest property retrieval method. We also apply a 2-D model of line of sight to the stems in the field survey data to quantify the relative roles of stem and non-stem sources of occlusion in limiting TLS line of sight.

By focusing on challenges and improvements to TLS forest sampling through the interlinked concepts of sample completeness and representativeness, we expand on the work of previous research on large-area forest surveying with TLS (Strahler *et al.*, 2008; Yao *et al.*, 2011; Abegg *et al.*, 2017; Wilkes *et al.*, 2017).

## MATERIALS AND METHODS

### Overview

We use a large number of TLS scans (761) from the Compact Biomass LiDAR (CBL) (Paynter *et al.*, 2016) within a large section of forest (255 650 m^2^) where every stem has been surveyed by traditional field methods: the Harvard Forest Global Earth Observatory (ForestGEO) plot in central MA, USA. We combine the TLS and field survey information to assess the availability of line of sight to stems by matching sets of points from TLS returns to the position of stems reported in the field survey. These TLS-observed stem sets are compared with two additional stem sets derived from the stem positions and sizes in the field surveys: stems expected based on the TLS range, and stems expected based on 2-D modelling of the TLS’s line of sight at each scan location. Comparisons are made between stem sets at each TLS scan location and between stem sets captured with each of three TLS sampling strategies (plot, transect and point sampling).

Stem counts from the stem sets were compared to separate the influence of occlusion from stems and from non-stem sources (other vegetation, other objects, and topography). We also used these stem sets to assess the influence of occlusion on the area-based property of stem density, and we evaluated alternative methods to derive the sampled area from the TLS scans. Finally, we compared these stem sets to uncover biases in the representation of various tree properties recorded in the field surveys, such as stem diameter and species.

### Field survey data

Field data were obtained for the 35-ha CTFS-ForestGEO site at the Harvard Forest and Long Term Ecological Research (LTER) site in Petersham, MA, USA (Anderson-Teixeira *et al.*, 2015). The ForestGEO site is a mixed temperate forest composed of a distinct set of forest structures and compositions. The upland areas are primarily dominated by *Tsuga canadensis* (eastern hemlock), *Acer rubrum* (red maple), *Quercus rubra* (red oak) and *Pinus strobus* (white pine) trees, while a lowland swamp to the northeast of the plot’s centre is dominated by *Ilex verticillata* (winterberry holly). Between 2010 and 2014, every woody stem in the ForestGEO site with a diameter >1 cm was documented in a dataset of 116 227 tagged stems (Orwig *et al.*, 2015). The 500 × 700-m area has been divided into a marked grid of 20-m plots, with a 10-m sub-grid. This extensive grid provided an ideal space to compare TLS with field survey data (Orwig *et al.*, 2018).

### TLS data

The CBL is a portable, rapid-scanning TLS (Paynter *et al.*, 2016) with a maximum effective range of 40 m. Its angular resolution is 0·25° and its beam divergence is 15 mrad, which is large compared with most other TLSs used for forestry. The CBL can register two returns from a single pulse, and all returns were retained here. Each scan was performed with the TLS levelled, aligned to true north, and placed in a known location in the ForestGEO site grid to enable co-registration with the ForestGEO field survey data. TLS scans were collected across a substantial portion of the ForestGEO site using three sampling strategies (plot, transect and point), but they avoided an impassable region of swamp near the centre of the site.

**Fig. 1.**
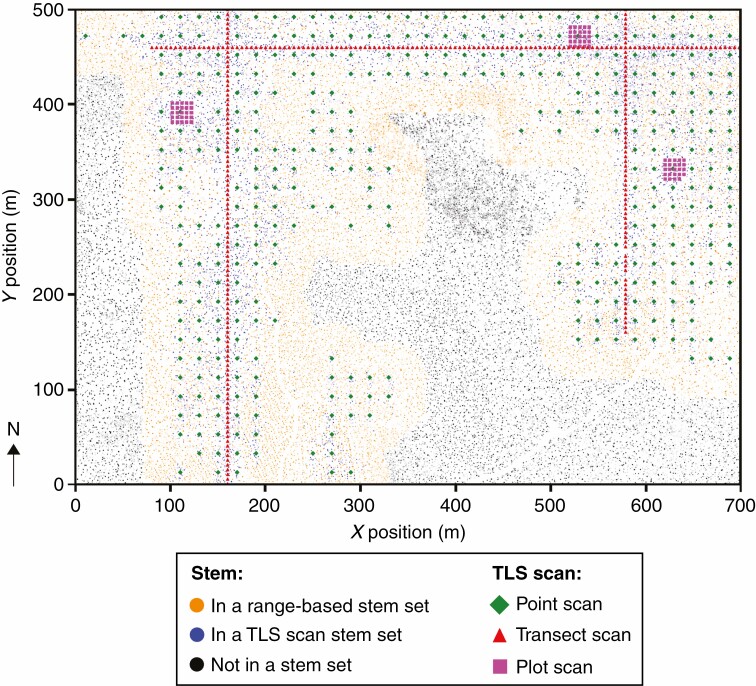
Map of the ForestGEO site with every stem plotted according to its position and DBH from the field survey data. The locations of all CBL TLS scans taken for this study are shown. Note the increased density of stems in the north-centre of the site, indicating the region of swamp area that was avoided during TLS scanning. Regions in the west and south-east were avoided due to their transition into distinctly different forest ecotypes.

The plot sampling was conducted in August 2017, and consisted of three 20 m × 20 m square plots, chosen at random from a section of the ForestGEO site grid, with each plot containing TLS scan locations on a 5-m-spaced grid (25 scan locations per plot). One scan failed, resulting in a total of 74 TLS scans between the three plots. The transect sampling was conducted in August 2017 and consisted of TLS scans spaced 5 m apart along two north–south transects and one east–west transect, chosen at random from the ForestGEO site grid (Fig. 1). There were a total of 294 transect scans. The point sampling was conducted in August 2016, and consisted of 393 scans, each at the centre of a square in the ForestGEO site grid. Point sampling represented an attempt to cover as much of the ForestGeo site as possible given the available resources (Fig. 1).

Additionally, another TLS instrument, the Leica BLK360 (angular resolution of 0·12°, beam divergence of <0·2 mrad) was used to scan nine locations within the area scanned by the CBL, in order to evaluate whether the wide beam divergence of the CBL was a controlling factor in the results. However, unless otherwise stated, any reference in this study to a TLS is to the CBL.

### Deriving range-based expected stem sets from field survey data

For each TLS scan location, we derived the complete set of stems that would be expected to be observed by a TLS if there was no occlusion to limit the line of sight, or any other limitations to the observation of stems. This expected stem set is simply every stem from the field survey data that was within the maximum range of the TLS (40 m), and therefore serves as the basis for comparison for the other stem sets. Field stems were filtered based on their position at the centre of their stem. Descriptions of results as overestimations or underestimations in this study are relative to this complete, expected, range-based stem set.

### Deriving line-of-sight-based stem sets from field survey data

**Fig. 2.**
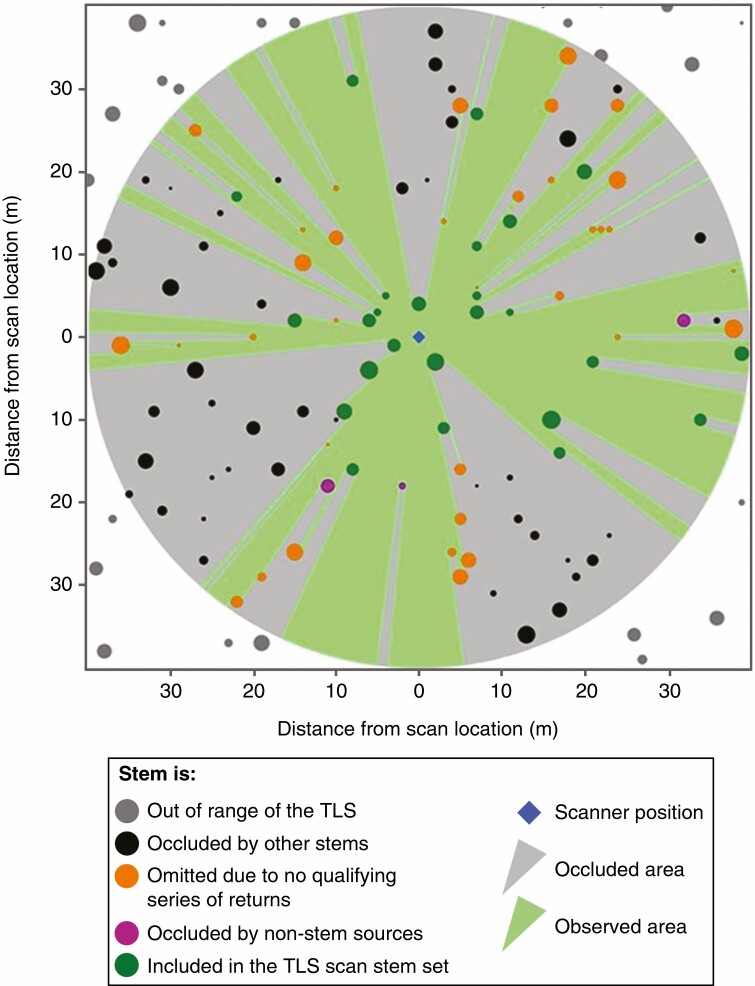
Demonstration of the sources of incompleteness in a TLS scan, and the factors influencing whether stems in the field survey data are present in the TLS scan stem sets. Note that this visualization has been modified to show the demonstrated principles clearly, and it is not a sample of real data. The stems are positioned according to a location from the field survey data, but their DBH has been doubled to make the patterns of occlusion easily visible. The patterns of occlusion are determined by the 2-D line of sight assessment of the field data used throughout this study. The stems that are omitted due to no qualifying series of returns (yellow circles) were selected in this visualization based on their narrow available lines-of-sight. The stems occluded by non-stem sources (purple) were randomly chosen from the stems to which line of sight was otherwise available.

We wanted to assess the role of stems in the restriction of TLS line of sight, relative to the role of non-stem sources of occlusion (other vegetation, other objects and topography). Therefore, for each TLS scan, we sought to establish the minimum effect of occlusion resulting from just the stems. This minimum effect of occlusion was derived using a 2-D model of line of sight from the TLS scan location to the stem locations recorded in the field survey data (Fig. 2). In this line of sight model, each stem was represented as a circle defined by its DBH recorded in the field survey data. In the model, a stem was occluded by line of sight according to its size and position in the field survey data relative to the TLS scan location. Therefore, the line-of-sight-based stem set for each TLS location consisted of the subset of stems from the range-based expected stem set that were not fully occluded by other stems according to this model.

### Deriving observed stems sets from the TLS scans and field survey data

We aimed to establish which stems each TLS scan had line of sight to. For each TLS scan, the stems that were potentially observed were the range-based stem set derived from the field data. To have observed a stem from this stem set, the TLS scan had to have at least one qualifying series of TLS returns within a 0·5-m radius of the location of the stem as at it was recorded in the field data. A qualifying series of TLS returns consisted of at least ten LiDAR returns, where all the returns originated from pulses with zenith (vertical) angles between −10 and +10° of the optical plane and were emitted during one rotation of the scanner’s mirror (so the azimuth angle of the pulses was similar). Additionally, each return in a qualifying series had to be within a 5-cm horizontal radius of its direct upward and downward neighbours, resulting in a more or less vertical stack. If multiple stems in the field survey data were within 0·5 m of a qualifying series of returns, the series was only used to suggest line of sight to the closest stem. Conversely, any number of qualifying series of returns were allowed to be attributed to a single stem in the field data. Any qualifying series of returns with no corresponding stem within 0·5 m were ignored, although such false positives were rare. Thus, the observed TLS scan stem set was guaranteed to be a subset of the range-based stem set from the field survey data, and was likely to also be a subset of the line-of-sight-based stem set.

It should be noted that this method was designed to interact with the detailed field survey data available for this study, and it should not be understood as an independent stem detection method. It is functionally a line of sight assessment. However, rather than simply use a single return in the vicinity of a recorded stem as evidence of line of sight, we have placed some constraints (i.e. the requirements for a qualifying set of returns) aimed at limiting the assessment to stem-like objects. Additionally, the use of the field survey data ensures that we are only looking for stem-like objects in locations where we know real stems to be. The mechanics of this approach are shown in detail in Supplementary Data Fig. S1, and the implications are further explored in the Discussion section.

### Comparing stem sets and assessing representativeness

We processed all 761 scans with the methods described above, ending up with TLS scans divided into three groups according to sampling strategy (plot, transect and point), and three sets of stems for each TLS scan location:

(1) The range-based stem set from the field survey data.
(2) The line-of-sight-based stem set from the field survey data.
(3) The TLS scan stem set from the combination of the TLS scans and the field survey data.

We then compared the stem counts and stem densities between these stem sets at each TLS scan location, and we compared the complete groups of stem sets from plot, transect and point sampling with the total population of stems within the study area. We define the population of stems in the study area as: all stems that were within a 40-m range from all scan positions (the union of all range-based stem sets, visualized as orange and blue stems in Fig. 1).

We also examined properties of the stems taken from the field survey data, namely DBH and species, to see if these properties influenced the frequency with which stems were observed in the TLS scan sets. As the basis for comparison, we used standard statistics of the stem properties (mean, median and standard deviation of DBH, for example).

We also employed Kolmogorov–Smirnov (KS) tests (Massey, 1951) to compare the distributions of stem properties in the different stem sets (expected, line of sight and observed) and sampling strategy groups (plot, point and transect samples) with the distribution of stems within the study area. Kolmogorov–Smirnov tests compare distances between the cumulative probability distributions of samples, with a significant result suggesting a difference between the distributions. In this study, we evaluated the *P* values from KS tests at an *α* value of 0·05.

We compared cumulative stem densities between TLS scan-, line-of-sight- and range-based stem sets. We conducted these cumulative analyses both for the transects, where scans were added in the order that they were conducted along the transect, and for the point scans, where stem sets from the scans were added to a group in a random order. For the point scans, 500 iterations of scan order were used. For each transect and each group of point scans, we extracted the cumulative stem sets as each scan was added (as the groups grew in size from 1 to *n* total scans). For each stem property at each group size of the randomly ordered iterations of point scans, we derived the 95 % confidence interval for the mean from a Student’s *t* distribution, the interquartile range, and the coefficient of variation from the 500 iterations. To analyse the representation of species between groups of stem sets in a cumulative manner, we used Simpson’s diversity index (Simpson, 1949).

We also applied a KS test to the complete distributions of various stem properties in the TLS and range-based stem sets to ascertain whether the overall distributions of observed stems differed significantly from the range-based, expected distributions.

When analysing the plot scan data, all of the stem sets were limited to the square boundaries of the intended plot. When analysing the transect scan data, we derived scan sets for both the 40-m maximum range of the scanner and for a 20-m range to evaluate the transect as a narrower corridor, maximizing the benefits of the considerable overlap between scans along the transect vector.

### Defining area estimation for stem density

**Fig. 3.**
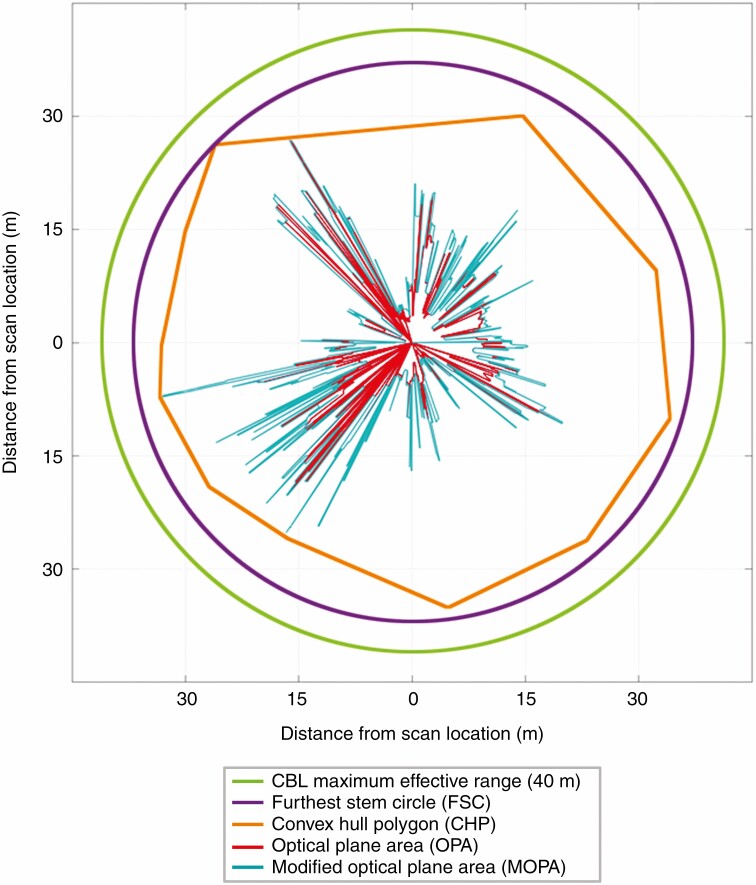
Diagram demonstrating the different definitions of area evaluated in this study for the retrieval of stem densities.

The default definition of area for calculating area-based metrics, such as stem density, is the area of the catchment zone for the sample. In the context of this study, the catchment zone for the stem sets was a circle defined by the effective range of the TLS (with the exceptions noted in the previous section). However, since occlusion blocks line of sight to stems, the stem densities resulting from this definition of area will always be underestimations. Here we evaluated four alternative definitions of area (Fig. 3) for stem density calculations that might better represent the area that was sampled, given the stems that were observed:

(1) Area of a circle whose radius is equal to the distance to the furthest stem observed by TLS at the scan location, referred to as the furthest stem circle (FSC).
(2) Area of a convex hull polygon (CHP) formed from the locations of the stems observed in the TLS scan stem set.
(3) Area of a polygon formed from the clockwise joining of the location of TLS returns from pulses emitted at the optical plane of the instrument, referred to as the optical plane area (OPA).
(4) Area of a polygon formed from the clockwise joining of the location of TLS returns that were within ±10 cm of the optical plane, referred to as the modified optical plane area (MOPA).

## RESULTS

### Stem counts

**Fig. 4.**
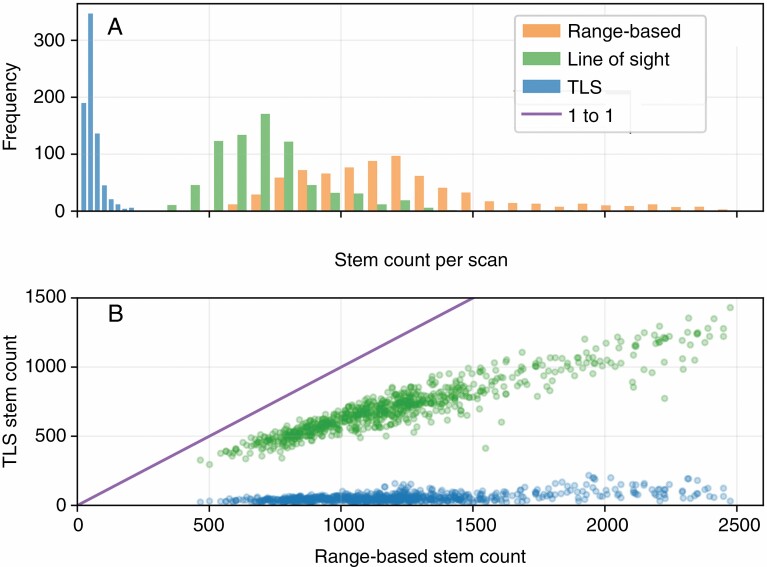
Stem counts for TLS scan stem sets. A histogram (A) displays stem counts from individual TLS stems sets and their line of sight, and range-based counterparts. A scatter plot (B) displays the relationship between stem counts from line of sight and TLS stem sets in comparison with their range-based counterparts.

Counts of stems in the TLS scan stem sets from the point and transect scans were far lower than the stem counts of their expected range-based and line-of-sight-based counterparts (Fig. 4, Table 1). The line-of-sight-based stem counts from the field survey data were also lower than their range-based counterparts, but the distributions still overlapped (Fig. 4A). However, the plot-based TLS and line of sight stem sets contained every stem present in their range-based counterparts for all three plots. The nine scans with the Leica BLK360 instrument resulted in a larger number of stems in the TLS scan stem sets (96 ± 25 stems per scan) than the CBL (58 ± 31 stems per scan).

**Table 1.** Mean stem count and standard deviation in real and simulated TLS scans and field data

	Range-based	Line of sight	TLS
Stem count (stems per scan)	1234 ± 453	716 ± 225	58 ± 31

The mean and standard deviation of the number of stems observed within 40 m of the 761 scan positions were calculated for individual range-based, line-of-sight-based and TLS stem sets. Stem counts represent the total number of stems observed from each position, allowing for repeated observations of stems from nearby scan locations.

Of the three sampling TLS methods tested, point sampling achieved the highest representation of stems in the study area (with 21 % of range-based stems observed) with the highest information gain per scan (39 unique observations of stems per scan; Table 2). Transect sampling had less coverage (12 %) and information gain (28 unique stems per scan) than did point sampling, but both point and transect sampling had a much higher coverage of the study area and information gain compared with plot sampling (0·3 % and 3 unique stems per scan; Table 2).

**Table 2.** Acquisition statistics of TLS sampling strategies

	Transect	Point	Plot
Total number of scans	294	393	74
Number of unique stems per scan	28	39	3
Total unique stems observed	8148	15 325	229
Stems observed in study area (%)	12	21	0.3

### Stem density

**Fig. 5.**
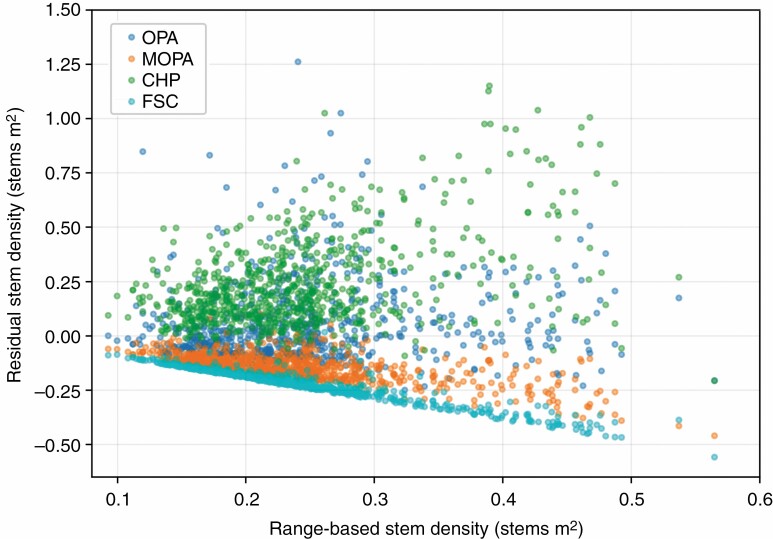
Residuals of TLS stem densities produced with four definitions of area in comparison with field stem densities produced with a range-based definition of area.

**Fig. 6.**
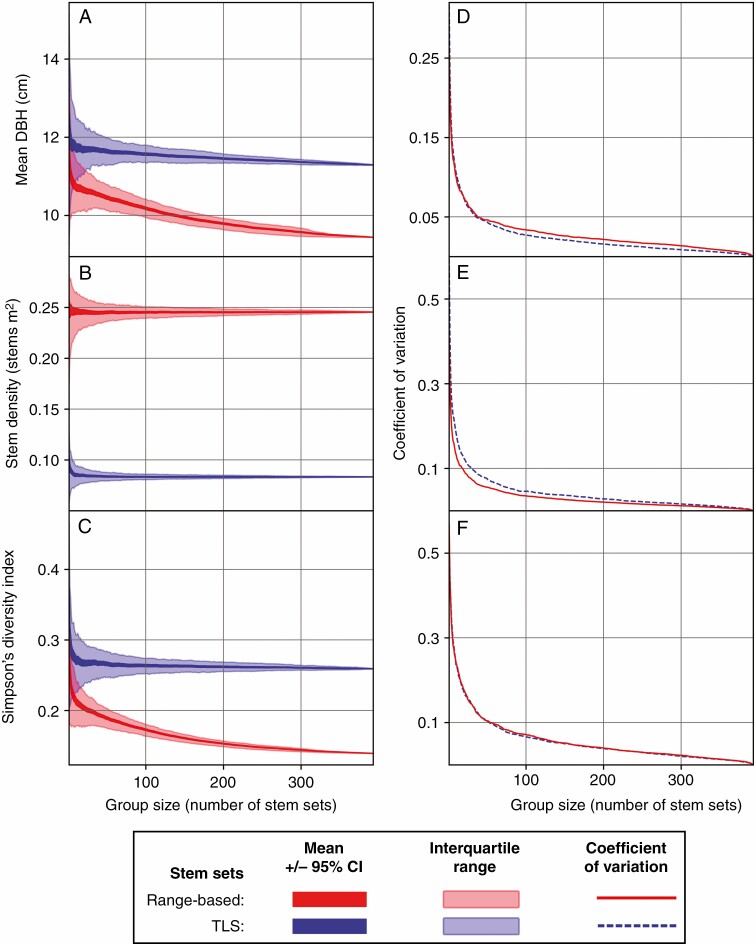
Confidence intervals, interquartile ranges and the coefficient of variation for the range-based and TLS stem sets from 500 random iterations of TLS scan locations from point sampling (*n *= 393 scans). The left column shows the 95 % confidence interval and interquartile range of cumulative mean DBH (A), mean stem density (B) and Simpson’s diversity index (C). The right column (D, E and F) shows the trend in the coefficient of variation for each metric.

Stem densities that were calculated by combining stem counts from TLS scans with an area defined by the maximum range of the TLS (40 m) were much lower than the baseline stem density from the field survey stem counts for the same area (RMSE = 0·06, *R*^2^ = 0·25). Defining the area for the TLS scan stem densities using FSC (RMSE = 0·06, *R*^2^ = 0·19) produced an overestimate of field stem densities, while CHP (RMSE = 0·09, *R*^2^ = 0·41) produced consistently lower estimates of stem density than the field survey (Table 3). However, defining the area by OPA (RMSE = 0·37, *R*^2^ = 0·02) and MOPA (RMSE = 0·03, *R*^2^ = 0·37) improved the relationship considerably, even though MOPA also produced underestimates. The residuals of all of the methods displayed heteroscedasticity, with larger residuals in areas of higher stem density (Fig. 5). Cumulative mean stem density, estimated by adding point scan stem sets in a random order to groups of increasing size (for 500 iterations), converged to a stable estimate of mean stem density in <50 scans, but TLS densities (calculated with MOPA) remained substantially lower than their range-based counterparts (Fig. 6).

**Table 3.** Linear regression fits and relative area statistics of TLS and range-based stem densities for the four area correction methods

	Optical plane area (OPA)	Convex hull polygon (CHP)	Furthest stem circle (FSC)	Modified optical plane area (MOPA)
*R* ^2^	0·02	0·41	0·19	0·37
RMSE	0·37	0·09	0·06	0·03
Mean normalized area	0·050	0·025	0·646	0·131
Standard deviation	0·024	0·007	0·269	0·046
Maximum	0·134	0·040	1·000	0·270
Minimum	0·001	0·007	0·035	0·022

Mean, standard deviation, maximum and minimum statistics were calculated from distributions of normalized areas, derived by dividing each area metric by a 40-m range-based circular area. The normalized area can be understood as a correction factor for the range-based circular area.

**Fig. 7.**
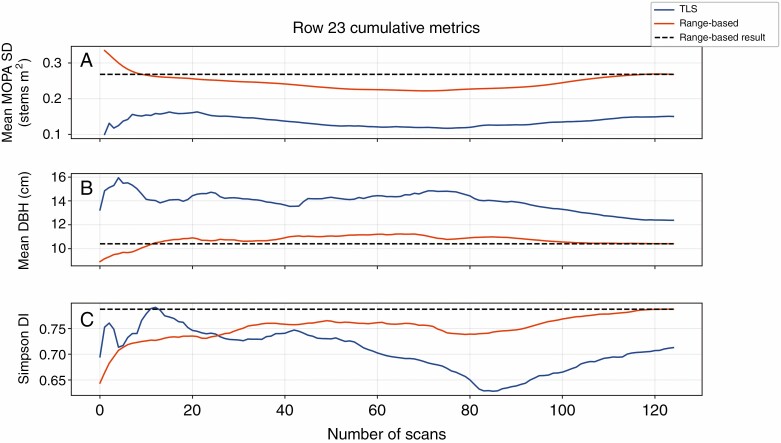
Cumulative mean stem density (SD) (A), mean DBH (B) and Simpson’s diversity index (DI) (C) for TLS and range-based stem sets from scan locations along the transect of row 23 of the ForestGEO plot.

**Fig. 8.**
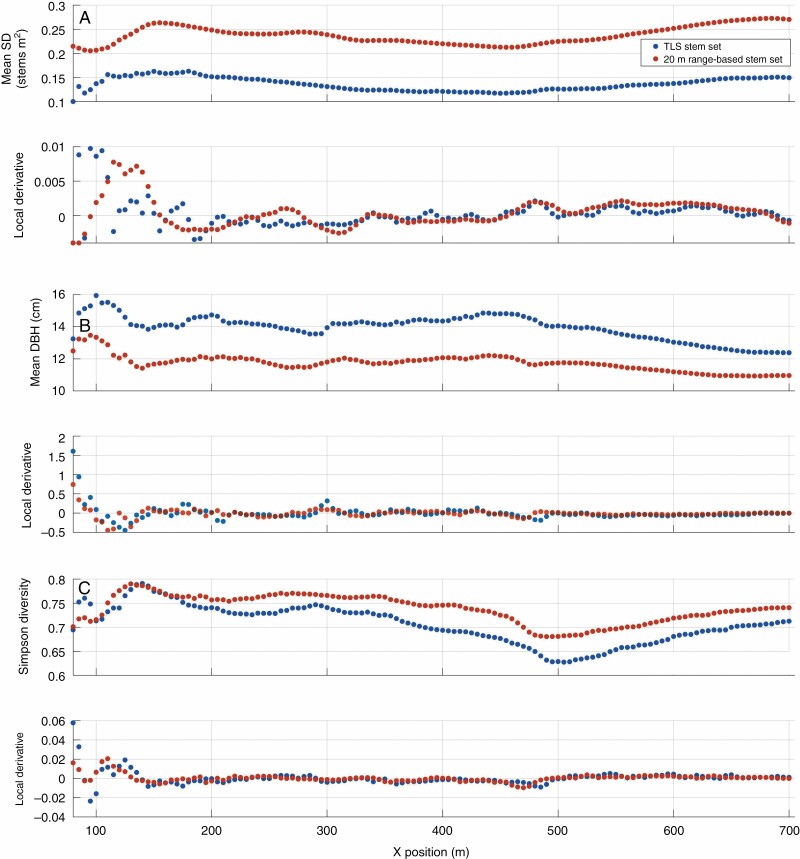
Cumulative mean stem density (SD) (A), mean DBH (B) and Simpson’s diversity index (C) for TLS and range-based stem sets, both limited to maximum ranges of 20 m, from scan locations the transect of row 23. For each metric, two plots are displayed, showing from top to bottom (1) a scatter plot of the cumulative metrics from TLS (blue) and range-based stem sets (orange) calculated at each position along the transect; and (2) a plot of the local derivative of the cumulative metrics of TLS (blue) and field (orange) stem sets.

Among the stem sets from the different TLS sampling strategies, mean stem densities from plot TLS scan stem sets (0·19 stems m^−2^) were the closest to the mean of the distribution of the range-based densities within the study area (0·25 stems m^−2^), although they still were an underestimation (Table 4). The TLS scan stem sets from the transect and point sampling strategies both greatly underestimated mean stem densities, but were closer to the standard deviation, maximum and range of their range-based distribution. Kolmogorov–Smirnov tests suggested that the distributions of stem densities from TLS stem sets and range-based stem sets were significantly different (*P* < 0·01) for each of the TLS sampling strategies (Table 5). For the transect scan stem sets, the cumulative mean stem density (accumulated one TLS stem set at a time, along the transect) covaried with the ranged-based stem set equivalent (Figs 7 and 8; Supplementary Data Figs S2 and S3), but remained lower throughout the transect.

**Table 4.** Comparison of TLS stem density statistics with the range-based stem sets by sampling strategy

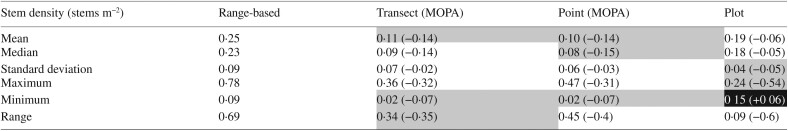

Transect and point stem densities were calculated using the MOPA area, while plot densities were derived from area of the plot square (20 m × 20 m). The value for each statistic is shown for each sampling strategy, accompanied by its residual from the range-based value in parentheses. For each statistic (row in the table), the value(s) with the highest magnitude negative residual is shaded grey and the value(s) with the highest magnitude positive residual is shaded black.

**Table 5.** Kolmogorov–Smirnov test results comparing the distributions of TLS and the study area stem sets

		Transect	Point	Plot
Stem density	*H*	1·00	1·00	^–^
	*P* value	<0·01	<0·01	–
	KS statistic	0·70	0·80	–
DBH	*H*	1·00	1·00	1·00
	*P* value	<0·01	<0·01	<0·01
	KS statistic	0·18	0·14	0·30

Distributions of stem density and DBH from each TLS sampling strategy are compared with distributions of the range-based stem set (for stem density) and the entire population of stems in the study area (for DBH). Plain text shows distributions that were significantly different (*H* = 1 and *P* < 0·05). Plot data were excluded from the stem density comparisons because of the small sample size (*n* = 3 stem densities). H denotes the rejection (1) or acceptance (0) of the null hypothesis that the distributions being compared are statistically similar.

### DBH from field survey data

**Fig. 9.**
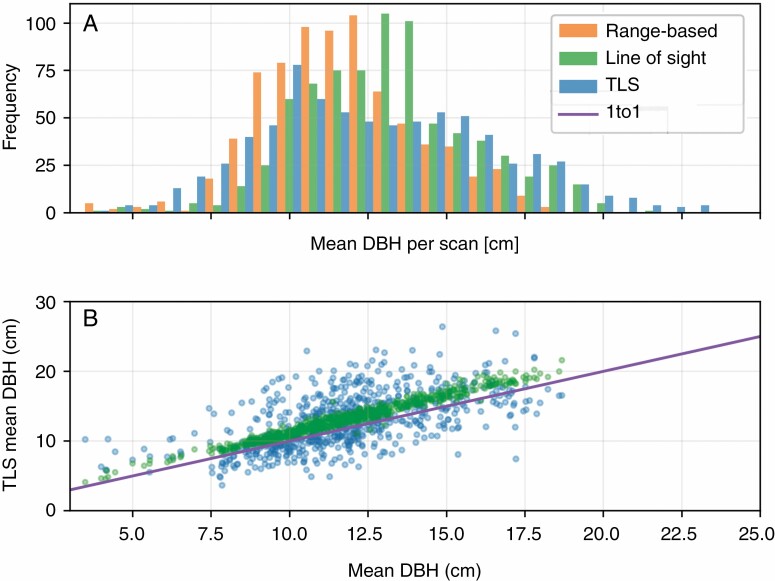
Histogram (A) and scatterplot (B) of the distribution of the mean DBH of individual range-based, line of sight and TLS stem sets.

The mean of the distribution of mean DBH values of the line-of-sight-based stem sets (13·1 cm) was higher than that of the range-based stem sets (11·7 cm), and the mean DBH of each individual line of sight stem set was higher than their range-based counterparts. The mean of the distribution of mean DBH of TLS stem sets (12·8 cm) was also higher than the range-based stem sets. However, the relationship of the mean DBH of the individual TLS scan stem sets to their ranged-based counterparts had generally high residuals and no clear overall bias (Fig. 9). Cumulative mean DBH for point scan stem sets were higher than their range-based counterparts, and they diverged with increasing numbers of scans (Fig. 6).

**Fig. 10.**
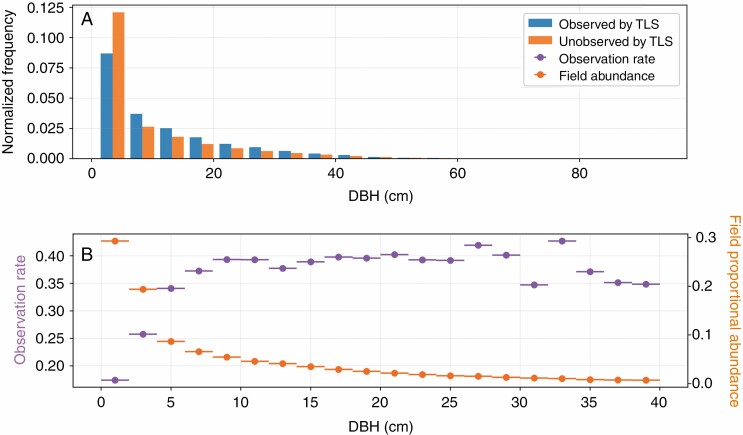
Histogram of the diameter of stems observed in TLS scan stem sets, and stems unobserved based on the corresponding range-based stems sets (A) and observation rate by DBH size (B). A 5-cm bin width was used to summarize the observation rate (purple, left axis), with bars representing the bin width (B). The proportional abundances of stems (orange, right axis) within each of the DBH size bins was calculated from the study area stem set.

In TLS scan stem sets, stems with a DBH >10 cm were observed more frequently than were smaller stems (Fig. 10), even though small stems were far more abundant, according to the field survey data (Fig. 10B).

Of the three sampling strategies considered, the point-sampling TLS stem sets were closest in mean, median, standard deviation, maximum, minimum and range of DBH in the study area stem set, with the transect-sampling stem sets being the second-closest (Table 6). However, KS tests suggested that the distributions of DBH from TLS scan stem sets and range-based stem sets were significantly different (*P* < 0·01) for each of the TLS sampling strategies (Table 5). For the transect scan stem sets, the cumulative mean DBH somewhat covaried with the 40 m ranged-based stem set equivalent, and remained higher throughout the transect (Fig. 7; Supplementary Data Figs S2 and S3), although with some initial pronounced variance, including several notable inflection points. When compared with the 20-m range-based stem sets, transect stem sets covaried even more with the field data, with similar rates of change and locations of inflection points along the vector of the transect (Fig. 8).

**Table 6.** Comparison of TLS DBH statistics with the study area stem set by sampling strategy

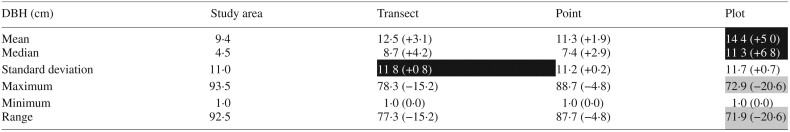

The value for each statistic is shown for each sampling strategy, accompanied by its residual from the range-based value in parentheses. For each statistic (row in the table) the value(s) with the highest magnitude negative residual is shaded grey, and the value(s) with the highest magnitude positive residual is shaded black.

### Species from field survey data

**Fig. 11.**
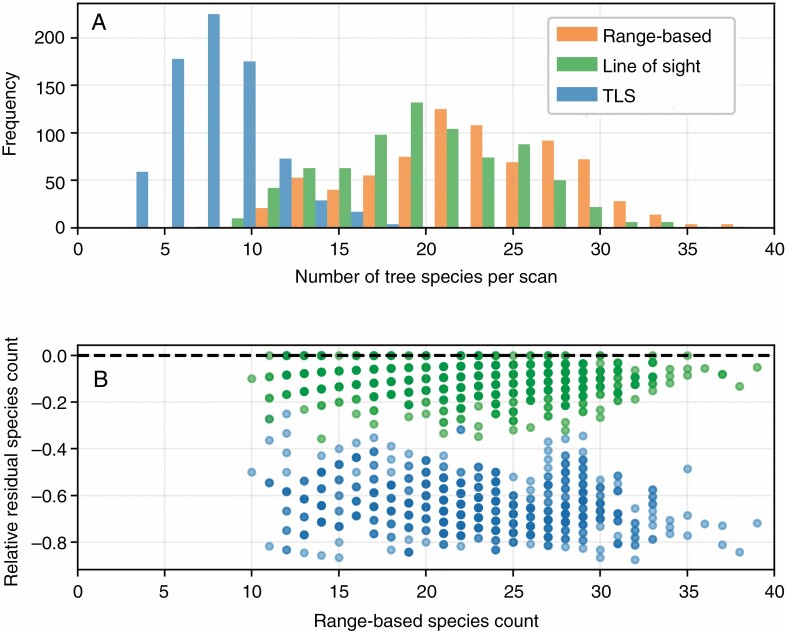
Number of tree species per scan identified in range-based, line of sight and TLS stem sets in a histogram (A), and a plot of the residuals (B) between TLS stem sets and their range-based counterparts (blue), and between line of sight stem sets and their range-based counterparts (green), normalized by the number of species in the range-based stem sets.

The line-of-sight-based stem sets had fewer species (20·2 ± 5·1 species) than their range-based counterparts (22·7 ± 5·5 species), but had a similarly shaped distribution (Fig. 11). In contrast, stem sets from TLS scans had much lower species counts (8·0 ± 2·8 species), with a substantially different distribution (Fig. 11). Cumulative Simpson’s diversity index for groups of TLS point stem sets was consistently higher than for their range-based counterparts, and stabilized after 50 scans were added to a group (Fig. 6E).

**Fig. 12.**
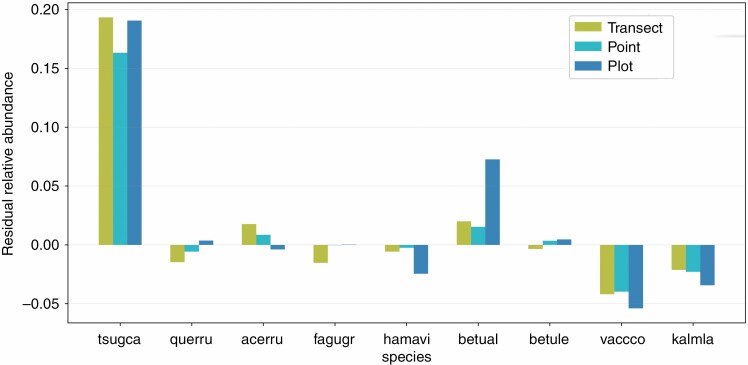
Residuals of relative species abundance for each TLS sampling strategy for the nine common tree species in the study area. Tree names are condensed to the first four letters of their genus and the first two letters of their species. For example, tsugca corresponds to *Tsuga canadensis*, commonly known as eastern hemlock.

Point and transect sampling strategies produced stem sets that included representatives of the majority of the 54 species in the range-based stem sets at least once (the point scan set included 43 unique species and the transect scan set included 40 species), whereas plot sampling, which has much lower total coverage of the ForestGEO site, only represented 19 species across its scan sets. Comparing the abundances for each species across the sampling strategies (Fig. 12) showed that the plot strategy was least similar to the range-based stem set abundances of certain species, such as yellow birch (*Betula alleghaniensis*). In contrast, point and transect strategies showed comparable residuals for most species, with transect abundances generally having slightly larger residuals than point abundances (Fig. 12). For the transect scan stem sets, the cumulative Simpson’s diversity index covaried with both the corresponding 40- and 20-m range-based stem sets, and was lower throughout most of the transect (Figs 7 and 8).

## DISCUSSION

### Completeness of TLS samples

Stem sets derived from combining TLS scans with the field survey data typically contained an order of magnitude fewer stems than both their range-based and line-of-sight-based counterparts, while the difference between the range and line of sight stem sets was much smaller (Table 1; Fig. 4). The line-of-sight-based stem sets factor in stem-based occlusion, whereas the TLS scan stem sets are subject to both stem and non-stem occlusion. Therefore, the discrepancy in the TLS scan and line-of-sight-based stem sets’ relationships to the range-based stem sets implies that non-stem sources of occlusion (other vegetation, other objects, such as rocks, and topography) are the major controlling influence on line of sight availability to stems for TLS scans. In the particular area of forest featured in this study, the prevalence of understorey vegetation explains this result, since other objects are rare, and topographical changes are typically gradual. However, we would expect any prevalent non-stem source of occlusion to have produced similar results. We would also expect non-stem occlusion to be a strong influence in forests of a similar ecotype to the ForestGEO site, and any other forest ecotypes with prevalent understorey vegetation, although these expectations warrant further study.

It is logical to assume that a technology that is limited to line of sight will not capture all objects from a set defined by predetermined spatial bounds. The prevalence of plot-based sampling strategies in TLS forest assessments (Yao *et al*., 2011; Newnham *et al.*, 2015; Wilkes *et al.*, 2017) suggests this assumption is widely held, as conducting a higher density of scans increases the chances of capturing all of the targeted objects within an intended sample area. The results of this study demonstrate how forming samples from multiple TLS scans that theoretically overlap in their coverage does improve the rate at which stems are included in TLS scan stem sets. However, even the most densely sampled plots are not guaranteed to be a complete sample if TLS scan positions are based on a predetermined layout, and we have shown here that sample incompleteness may have substantial impacts on sample representativeness (Figs 4, 5, 9 and 11).

One approach to avoiding TLS sample incompleteness for a plot-based sampling strategy would be to monitor the accumulation of information during the acquisition of scans. As each scan is completed, the progress towards completing the sample (as defined by the information requirements of the property of interest) would be updated. The sampling effort would continue to be expanded with additional scans, guided by the areas of missing information, until the plot sample was complete. A responsive, adaptive approach could also be applied to transect sampling to dynamically space the scans. For point sampling, monitoring the rate of change in the cumulative estimates of stem properties (by the coefficient of variation, for example) could provide an indication of when estimates have stabilized.

It was evident that sample completeness has particular implications for area-based metrics such as stem density. If the definition of area for the calculation of stem density is that of the predetermined space of a plot, or of the theoretical coverage of a TLS based on its effective range, then failing to capture every stem within that area results in an underestimation of stem density. This underestimation arises from the lower numerator of stem count being divided by the fixed denominator of intended sample area. Therefore, we investigated alternative methods to define area that would be more representative of the actual area sampled by the TLS while it was observing the stems in the resulting TLS scan stem sets. Of the definitions of area evaluated in this study (which also included FSC, CHP and OPA), MOPA was the most successful in reducing the discrepancy and eliminating the bias between stem densities from TLS stem sets and their range-based counterparts (Table 3; Fig. 5). We do not consider this study an evaluation of MOPA as an operational method, but we do consider deriving *a posteriori* estimates of sampled area from TLS scans a promising avenue for further development.

### Representativeness of TLS samples

The aim when sampling a subsection from a wider area of interest is that the subsection is a representative sample of the wider area. In terms of this study, the aim of any given TLS scan or group of TLS scans was to provide an estimate of stem properties that was useful in predicting the stem property at the scale of the ForestGEO site. From this definition, it can also be understood that sample completeness, discussed in the previous section, is functionally an evaluation of how representative the TLS scan and line-of-sight-based stem sets were of their ranged-based counterparts.

Generally, the more samples that are drawn from a population (a higher sample size), the more closely the statistics of that group of samples will resemble the statistics of the population (increased representativeness). This tendency was reflected in the improvements in the representativeness of the cumulative estimates of stem density, mean DBH and Simpson’s diversity index in the transect and point scans (Figs 6–8). The close tracking of derivatives between the TLS scan stem sets and their 20-m range-based counterparts (Fig. 8) showed that the TLS scan stem sets were representing the localized changes (within a 20-m radius) in the stem properties very well, if not their absolute values. Consistent biases in the retrieval of forest properties, with stable absolute or proportional offsets, could be ameliorated with correction factors established in future studies. However, given that such correction factors would be specific to the forest property, forest ecotype and TLS, this approach is far from ideal.

We did observe a tendency for smaller stems (<10 cm DBH) to be underrepresented in the TLS scan stem sets (Fig. 10), which persisted regardless of the sampling strategy (Table 6) or number of scans utilized (Fig. 6). These small size classes account for a large proportion of the population of stems in the ForestGEO site (Fig. 10B), and so this tendency to underrepresent them could have important implications for ecological studies. However, larger size classes are generally the most important to commercial forest applications, such as timber inventories, and these size classes (>10 cm DBH) appear to be detected at a relatively stable rate.

The variation and extremes of the DBH distribution within the study area (standard deviation, minimum, maximum and range) were not well represented in the plot scan stem sets, but were represented more closely in the transects and point scans. This lack of representation for the extremes of a stem property is unsurprising given the plots’ low overall coverage of the study area stems (0.3 %, Table 2), which themselves cover the majority of the ForestGEO site (Fig. 1). Since plot-based strategies require a relatively high level of TLS scanning effort, in terms of scans per unit of area covered, their coverage will tend to be lower than transect-based or point-based sampling strategies. However, for sites where field survey data are available, there is the option to employ stratified placement of plots (still with the benefits of being *a priori* and random) to mitigate limitations in representativeness. Remote sensing observations with wider coverage, such as from airborne (Cook *et al.*, 2013) and satellite (Dubayah *et al.*, 2020) instruments, might also offer an avenue to stratifying TLS sample placement.

Similar to the extremes of quantitative stem properties, and based on the same principle, representing species and their relative abundances also benefits from increased coverage of the wider area of interest. The representativeness of relative abundances (Fig. 12) was highest in point and transect scan stem sets. Again, this outcome is unsurprising, as covering more ground within an area of forest will increase the chance of encountering the variety of stems contained within it.

Transect sampling may provide a balance between the completeness of plot sampling and the representativeness of point sampling (Table 2). It should be noted that, even though a larger total number of transect and point scans were available, cumulative estimates of stem properties from their TLS stem sets tended to stabilize rapidly, often within 50 scans for point scans (Figs 7 and 8).

### Evaluation of range-based stem sets

The range-based stem sets were derived by sampling the field survey data according to the location of the TLS scans, and the maximum effective range of the CBL TLS. The use of 40 m as the maximum range of the CBL is conservative compared with manufacturer estimates, and the CBL has been demonstrated the ability to consistently detect objects up to this range (Paynter *et al.*, 2016), suggesting it appropriately parameterized for this study.

The precision and accuracy of the field survey data could have impacted the derivation of the TLS stem sets via the matching of field stems to TLS returns. During the ForestGEO site survey, the position of stems was recorded to the nearest vertex on a 0.1-m grid (Orwig *et al.*, 2015). This precision results in a potential positional error of 0.07 m for the location of a stem. Additionally, the recorded position for the stem is for the stem centre, and the LiDAR returns will correspond to the exterior of the stem, resulting in a discrepancy equal to the radius of the stem between the field survey stem location and the LiDAR returns. Furthermore, the CBL has a ranging accuracy of ±0.03 m (Paynter *et al.*, 2016). Therefore, without considering field survey operator error, the worst-case scenario for positional error for a stem of 0.6 m DBH (a large stem in the context of this study) is ~0.4 m. Since the matching process allows for up to 0.5 m between a qualifying series of LiDAR returns and a stem, even when other sources of error are at their most extreme and covaried, field survey operator errors of up to 0.1 m could occur before a 0.6-m DBH stem would fail to be matched to corresponding LiDAR returns. Based on these estimated tolerances, the stem-matching process is unlikely to have been frequently confounded by field survey operator error in this study. However, every effort should be made to minimize field survey errors, and operator error should be estimated empirically in future studies.

### Evaluation of line-of-sight-based stem sets

The line-of-sight-based stem sets were derived by modelling the 2-D patterns of occlusion when each stem in the field survey data was represented by a circle at its recorded position, and with a diameter equal to its recorded DBH. As stated in the methods, this was intended to be a representation of the minimum occlusion that would result from stems, providing a baseline to assess non-stem sources of occlusion when compared with stem sets from TLS data. While it is not a 3-D model of occlusion, assessing line of sight based on stem positions and DBHs in the field survey data is directly relevant to deriving observed stems sets from the TLS scans. The positions of stems in the field survey data are recorded at the height where DBH was taken for the stem, so leaning or convoluted stems are not a challenge to this 2-D assessment of line of sight.

In terms of being a good representation of TLS line of sight, the height of the optical plane of the TLS was set to the height at which DBH was recorded for the field survey (1.3 m). However, topography will disrupt the relationship between TLS optical plane height and stem DBH height. While topographical changes are mostly gradual in the ForestGEO site, this cannot be ruled out as an influencing factor in this study, but the fact that all of the TLS scan stem sets were subsets of their line of sight counterparts is reassuring.

The line of sight modelling approach does not consider the specifications of TLSs. The approach characterizes line of sight availability, but many TLSs used in forestry emit discrete pulses spaced at regular intervals of angle, or at least functionally have an angular resolution. Therefore, TLSs may not observe a particular line of sight to a stem even if it is available. Furthermore, the line of sight assessment does not consider TLS beam divergence, which increases the footprint of real TLS pulses over distance. However, predicting whether a particular TLS would record a return from an object is complex, even given knowledge of the relative location of the object and the line of sight to it. Since this study is not an evaluation of the performance of the CBL, and since the beam divergence of the CBL is much wider than other TLS used for forestry, a more general characterization of available line of sight seemed appropriate for this study.

The line of sight stem sets are directly impacted by errors in the field survey data. A missing, false, or mispositioned stem could change the predicted line of sight patterns quite considerably, and the detrimental effects on the accuracy of the resulting stem sets would be particularly exacerbated when the stem is larger or closer to a scan position, since it is then responsible for a larger region of occlusion. The impact of the accuracy of stem presence and position in the field survey data would also be greater in areas with higher stem densities, where a smaller change in the line of sight patterns would tend to result in a larger change in the membership of the resulting line of sight stem sets. Finally, the impact of stem presence and position accuracy would also vary with the size of other stems in the plot. Larger stems require a wider angular range of occlusion to fully occlude and are therefore more prone to false inclusion in line of sight stem sets, whereas smaller stems require a narrower angular range of occlusion to fully occlude and are therefore more prone to false exclusion from line of sight stem sets. Since the interactions between field survey operator error and the stem matching process and line of sight assessments are complex and potentially highly detrimental, it reinforces the necessity for field survey errors to be minimized and estimated empirically in future studies.

### Evaluation of TLS scan stem sets

The TLS scan stem sets were formed by matching qualifying sets of TLS returns with the position of stems recorded in the field survey data. In addition to the previously discussed considerations of the accuracy of the field survey data, the requirements to form the sets of TLS returns and the parameterization of those requirements also influenced the results of this study. The concept that a stem would be represented by a more or less vertical (5-cm tolerance between neighbours), unbroken series of returns from consecutive seems logical, and appeared functional in preliminary assessments, but it would not be suitable as an independent stem detection method without a structured and robust evaluation, which is not attempted in this study.

This study’s approach to assessing whether TLS scans had line of sight to stems is strictly guided by the use of the detailed field survey data of the ForestGEO site. The field survey data prevent sets of TLS returns from producing false positives (stems that did not exist) by discarding the (admittedly rare) qualifying sets of TLS returns that were not within 0.5 m of a field survey stem location.

We could have employed less strict requirements for qualifying series of TLS returns as potential observations of stems, even going as far as to declare any TLS return in the vicinity of a stem as evidence of line of sight to the stem. However, we chose to impose some constraints to limit the observations to sets of returns that are least stem-like in structure, since non-stem objects with vertical, flat surfaces are extremely rare in the ForestGEO site.

According to the most literal definition of having line of sight to a stem, the TLS scans certainly had line of sight to more stems than are represented in the TLS scan stem sets. If we were to loosen the requirement for observation of a stem yet further, to the observation of any portion of the structure of the tree associated with a stem, then the TLS would undoubtedly have had line of sight to a far higher proportion of the stems in the samples. Therefore, the findings of this study should not cast doubt on the efficacy of TLSs as a tool for forest inventory and ecological assessment. However, even our requirements for qualifying series of returns are not a guarantee that the TLS has captured the structural information necessary to retrieve particular stem properties. This study highlights the importance of evaluating and establishing the information requirements for retrieving properties of interest in advance of attempting to assess a particular forest site with TLSs.

As previously noted, the CBL has a lower angular resolution (0·25°) and a wider beam divergence (15 mrad) than most TLSs used operationally for forest assessments. Additionally, the CBL only records the first and second returns in each pulse (Paynter *et al*., 2016). This aspect of the CBL, coupled with its wide beam divergence, leads us to expect a much stronger range dependency in the representation of stems by returns in CBL data compared with other TLS.

To investigate the impact of the CBL’s specifications, nine locations within the study area were scanned with the Leica BLK360 instrument (angular resolution 0·12°, beam divergence <0.2 mrad) within the area sampled with the CBL, resulting in a larger number of stems in the TLS scan stem sets (96 ± 25 stems per scan) than the CBL (58 ± 31 stems per scan). Yet the Leica BLK360 scan stem sets still contained fewer stems than their line-of-sight-based and range-based counterparts by an order of magnitude, leading us to believe that the specifications of the CBL are not the most major influence on the results of this study. However, we still intend to conduct future studies of this type with a TLS of more representative specifications for the wider field of TLS forest assessment.

### Scope of inference of the study

This study sought to investigate whether more widely spaced, single-scan, point samples could be a useful tool for forest studies, but the scanning locations in this study were chosen non-randomly within the ForestGEO site. An initial set of scans that inspired the study were collected in service of a separate study of one particular species of tree (eastern hemlock), and even when many additional sampling locations were added for this study we still excluded the sizeable swamp in the centre of the ForestGEO site, and the western and south-eastern regions that are of distinctly different ecotypes (Fig. 1). Therefore, the results of this study do not include the full ForestGEO site in their scope of inference. If we return to the ForestGEO site for future studies, we will aim to expand the extent of the sampling to additional forest ecotypes.

As previously noted, this study is not an evaluation of the CBL, or TLSs in general, for retrieving any particular forest property. In addition, some of the findings of this study, in particular the dominance of non-stem sources in occlusion, may not hold for other forest types where understorey vegetation is sparser. Expanding this type of investigation into other forest types with similarly detailed field survey data would be of interest for future studies.

### Conclusions

In this study, we addressed two interlinked concepts in the use of TLSs for forest surveys and ecological assessment: the completeness and the representativeness of a TLS sample for a wider area of interest. We examined the influence of the defining characteristic of TLSs – limitation to line of sight observations – on the sets of stems observed by a large number of TLS scans. These TLS scans were taken in an area of forest where the position, size and other properties of every stem were known: the ForestGEO site (Petersham, MA, USA). We established that these samples can be understood to be incomplete if there is a discrepancy between what is expected to have been observed and what was observed, and we demonstrated the negative impact of sample incompleteness on sample representativeness. Most notably, the use of definitions of area that do not reflect the actual area that was sampled results in poor representations of area-based metrics such as stem density.

Although dense sampling strategies, such as the plot-based scanning grids examined in this study, have a great deal of potential redundancy in their coverage, they are still not guaranteed to produce complete samples, and also use a large amount of sampling effort for relatively small coverage of a wider area of interest. Transect and point sampling strategies may offer better representation of some stem properties for a similar sampling effort, thanks to greater coverage of an area of interest, but this study did not focus on the information requirements for retrieving any particular stem property directly from TLS scans. Thus, we recommend that alternative sampling strategies should continue to be considered, as they could provide better value in terms of information density and quality per unit of sampling effort, particularly as TLS technologies develop further.

The availability of the detailed field survey data allowed us to attribute a portion of the observed influence of occlusion directly to stems, and to conclude that non-stem sources of occlusion (such as other vegetation, other objects, and topography) were dominant in this forest. Viewed from another perspective, the prevalence of non-stem occlusion means that even extremely detailed stem survey data would not have allowed us to prepare an effective set of TLS scanning positions *a priori*, no matter which overall sampling strategy we had employed. Therefore, we see it as a necessity to develop the technology and protocols to monitor the information collected during TLS surveys while they are in progress. Then, we can adapt TLS sampling strategies on the fly, to ensure the information requirements to retrieve the forest properties of interest are met.

## SUPPLEMENTARY DATA

Supplementary data are available online at https://academic.oup.com/aob and consist of the following. Figure S1: workings of the stem observation method. Figures S2 and S3: cumulative moving means for all three transects and point scans.

mcab073_suppl_Supplementary_Figure_S1

mcab073_suppl_Supplementary_Figure_S2

mcab073_suppl_Supplementary_Figure_S3
