# Supplementary figures and images for: Sampling forests with terrestrial laser scanning
Source: Ann Bot. 2021 Jun 10;128(6):689–708. doi: 10.1093/aob/mcab073 (PMC8557379; doi:10.1093/aob/mcab073)

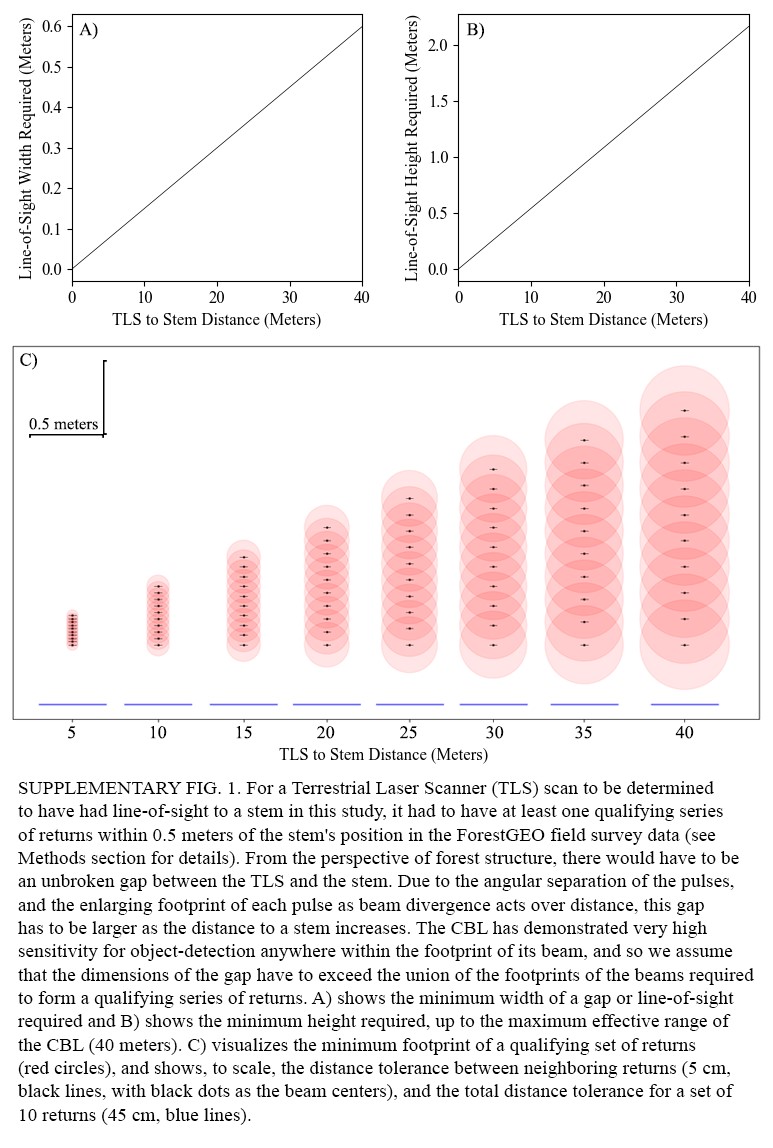

Supplement: mcab073_suppl_Supplementary_Figure_S1 [file mcab073_suppl_supplementary_figure_s1.jpeg]

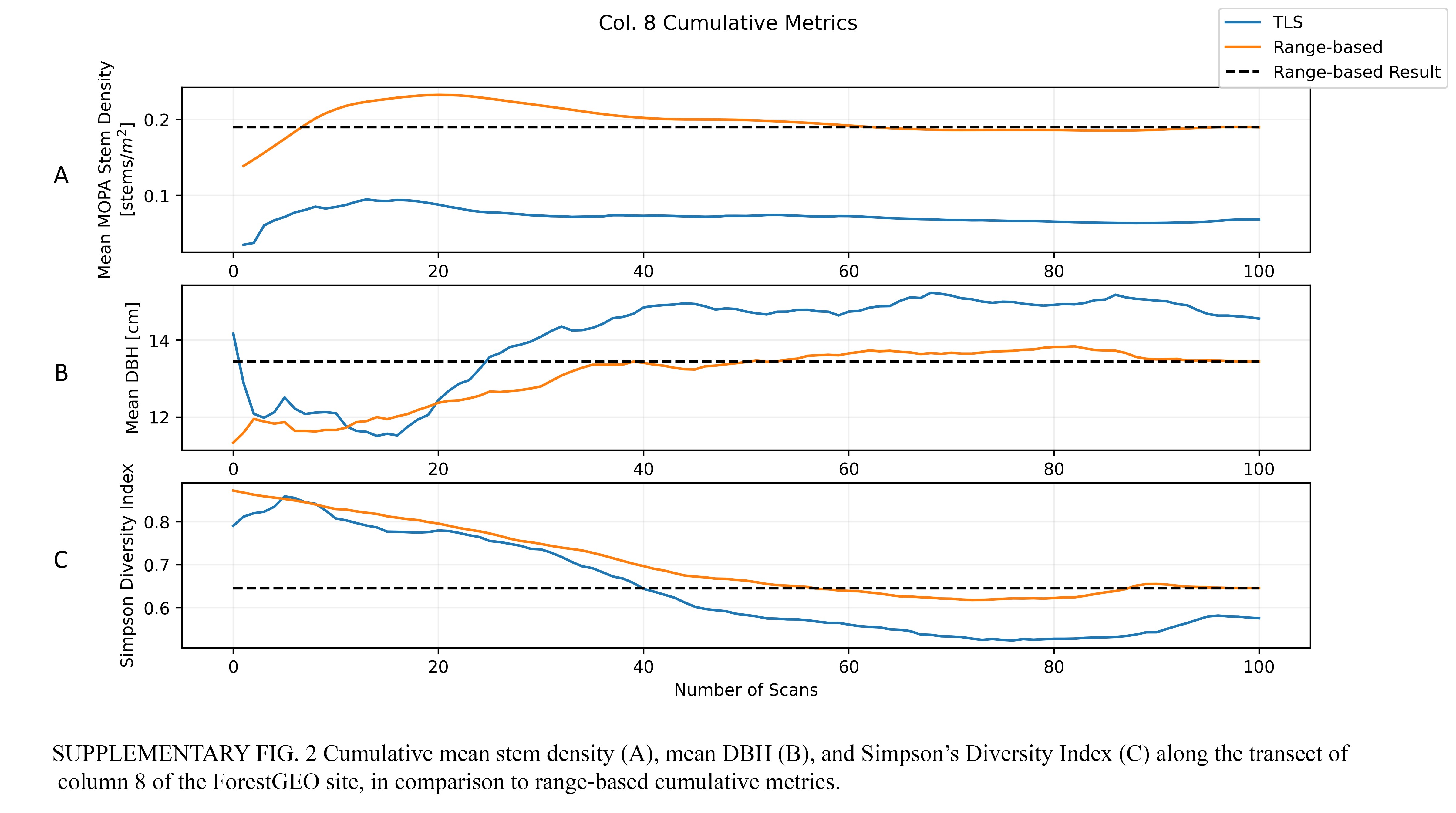

Supplement: mcab073_suppl_Supplementary_Figure_S2 [file mcab073_suppl_supplementary_figure_s2.jpeg]

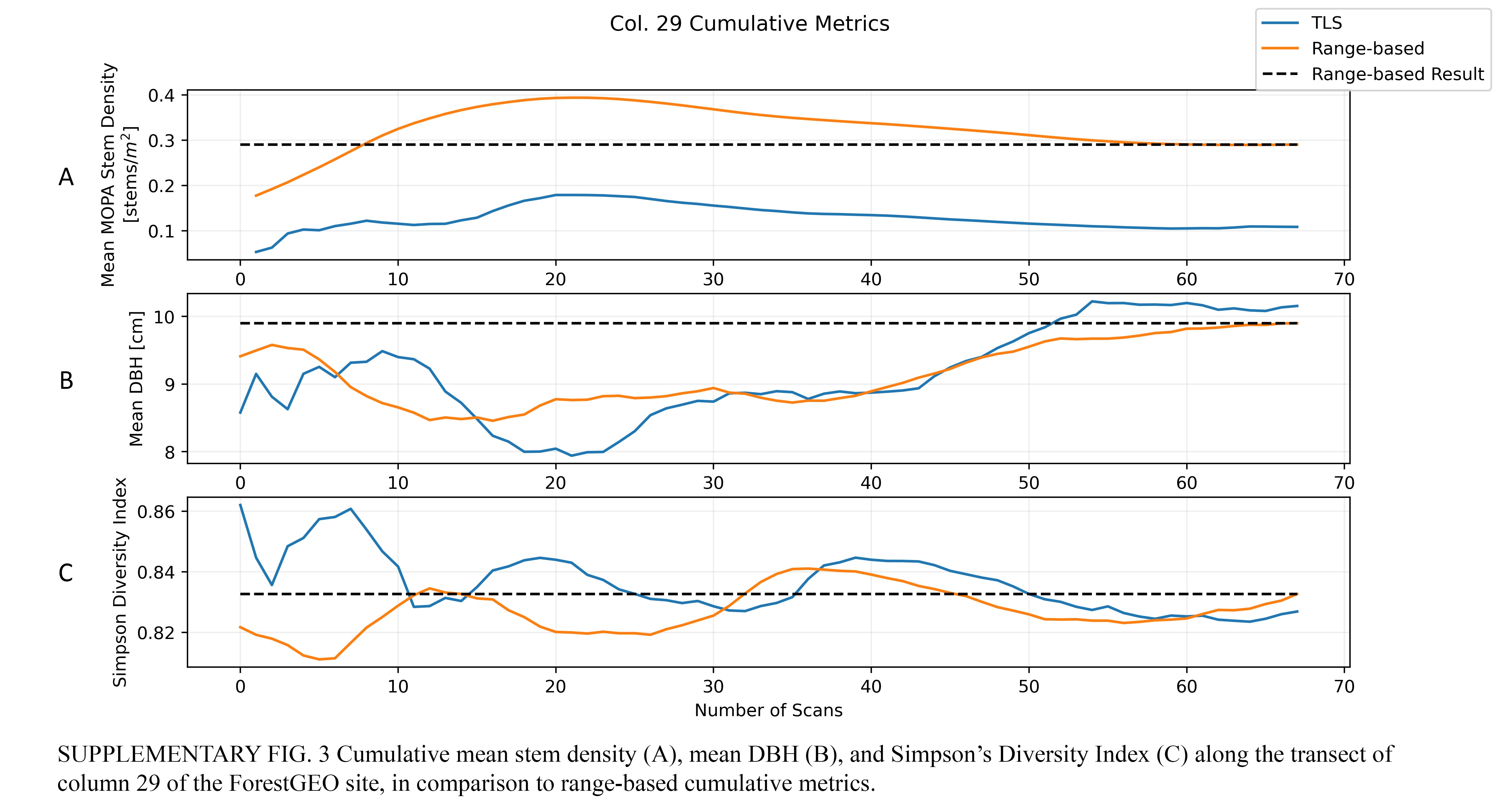

Supplement: mcab073_suppl_Supplementary_Figure_S3 [file mcab073_suppl_supplementary_figure_s3.jpeg]
